# MFA: A Smart Glove with Multimodal Intent Sensing Capability

**DOI:** 10.1155/2022/3545850

**Published:** 2022-07-11

**Authors:** Hongyue Wang, Zhiquan Feng, Jinglan Tian, Xue Fan

**Affiliations:** ^1^School of Information Science and Engineering, University of Jinan, Jinan 250022, China; ^2^Shandong Provincial Key Laboratory of Network Based Intelligent Computing, University of Jinan, Jinan 250022, China

## Abstract

At present, virtual-reality fusion smart experiments mostly employ visual perception devices to collect user behavior data, but this method faces the obstacles of distance, angle, occlusion, light, and a variety of other factors of indoor interactive input devices. Moreover, the essence of the traditional multimodal fusion algorithm (TMFA) is to analyze the user's experimental intent serially using single-mode information, which cannot fully utilize the intent information of each mode. Therefore, this paper designs a multimodal fusion algorithm (hereinafter referred to as MFA, Algorithm 4) which focuses on the parallel fusion of user's experimental intent. The essence of the MFA is the fusion of multimodal intent probability. At the same time, this paper designs a smart glove based on the virtual-reality fusion experiments, which can integrate multichannel data such as voice, visual, and sensor. This smart glove can not only capture user's experimental intent but also navigate, guide, or warn user's operation behaviors, and it has stronger perception capabilities compared to any other data glove or smart experimental device. The experimental results demonstrate that the smart glove presented in this paper can be widely employed in the chemical experiment teaching based on virtual-reality fusion.

## 1. Introduction

Many secondary school chemistry experiments currently contain damaging, costly, and sometimes dangerous experimental qualities, preventing students from doing practical operations for many of them. Simultaneously, because teachers have a finite amount of teaching energy, some students are prone to overlooking the most important aspects of experimental operation and exhibiting irregular operation behavior when conducting experiments. As a result, developing an intelligent, operable, and low-risk experimental platform system for secondary school experiments has become a pressing issue.

Three popular and effective technical solutions are available. The first is to create a virtual experimental platform [1] using virtual modeling software, which solves the problem of high costs and risks associated with teaching real experiments. However, most existing virtual experimental platforms use a mouse, a keyboard, and a monitor for experiments, reducing the user's sense of operation significantly. The second is the use of virtual-reality (VR) and augmented reality (AR) technologies, which allows people to have the same experience as if they were participating in a real experiment but require more memory. The third is to build a virtual-reality fusion experimental platform [2]. Because the existing platform relies on several cameras or indoor interactive input devices like KINECT to monitor the user's actions, issues like occlusion and the inability to observe minor experimental phenomena are common.

In light of the shortcomings of the previous three technical solutions, this paper finds that just one or two sensors can be employed to detect the user's hand position and movement in the study of the smart glove. As a result, this paper designs a smart glove and a mixed reality (MR) experiment system that can fuse multimodal data and, using a multimodal fusion algorithm, determine the user's experimental intent and direct the experiment, as well as correct and remind the user of any incorrect or unsafe procedures. The experimental intent of this paper refers to the experimental steps of user operation. By obtaining the real-time experimental intent of the user, the smart glove system can advise the user in time or correct and remind the user of incorrect and harmful steps. And the foundation for completing the experiment is understanding the user's experiment intent.

Therefore, the following are the primary innovations in this paper:

1. This paper designs a smart glove system based on MR experiment teaching, to overcome the limits of existing interaction devices and tools. Using multisensing fusion technology, the smart glove can complete complex user experiment intent analysis using only some simple sensors. In this study, a monocular camera is integrated at the wrist of the smart glove to solve the problems of visual occlusion and observation of subtle phenomena. Not only can the device capture data about the user's behavior throughout the experiment, but it can also detect the user's experimental intent and demand for the experimental scene.
2. Under the background of intelligent experiment, this paper proposes a multimodal fusion algorithm (MFA) which fuses the user's experimental intention in parallel at the intention level, and solves the limitations of the traditional multimodal fusion algorithm (TMFA) which is used to analyzing the user's behavior in serial. After acquiring the intent probability in the user's voice, visual, and sensor channels, this paper employs information weight method to fuse the user intent probabilities. The essence of MFA is to convert the user's abstract intent which is difficult to understand into a calculable probability problem.

This paper is organized as follows.  Section 2 comprehensively discusses related work.  Section 3 describes the prototype design of smart glove and construction of MR virtual-reality fusion laboratory.  Section 4 introduces the overall framework and MFA.  Section 5 analyzes and discusses the experimental results. Conclusions are presented in Section 6.

## 2. Related Work

### 2.1. Experimental Teaching of Virtual-Reality Fusion

The virtual-reality integration experiment is an experiment in which users interact with the virtual world using real-world experimental objects to interact with the virtual objects in a computer-simulated virtual environment.

Virtual experiments were first proposed by William Wolfe in 1989, and as computer multimedia graphic picture technology advanced, virtual experiments were gradually included in educational training. At the beginning of the development of virtual experimental teaching mainly based on Web technology, Aljuhani et al. [3] developed a virtual laboratory platform based on the Web platform, and users can conduct virtual experiments using a mouse.

Virtual-reality technology has progressively become popular as research progresses. Bogusevschi et al. [4] used virtual-reality technology to recreate the water cycle system in nature, and students were able to observe and study according to the program's principles. Salinas and Pulido [5] used AR technology to create a virtual platform to help students better understand conic curves. With the advancement of AR technology, the use of MR technology for teaching and learning has entered our vision. MR is a computerized virtual-reality technology that allows the real and virtual worlds to be presented and interacted within the same visual area. Lenz et al. [6] designed an MR speech lab that combines real and virtual classrooms, which can realistically portray the number of students and other noises that may be generated, while the teacher simulates daily teaching in a virtual environment using MR displays. Hu et al. [7] proposed a vision-based dynamic head posture tracking system, which improved the driving simulator's immersion and engagement.

In comparison with genuine trials, the virtual-reality fusion experiment not only increases users' interest in learning and aids their comprehension of knowledge, but also reduces consumables and risks. Virtual-reality fusion studies, as opposed to virtual experiments that divide the real from the virtual, provide an interactive feedback pathway between the virtual world, the real world, and the user, increasing the realism of the user experience. Researchers are gradually mixing MR technology with experimental education instruction, and MR technology has therefore become a popular study direction because it possesses the features of virtual-reality fusion and real-time interaction.

### 2.2. Understanding the Intent of Multimodal Fusion

Originally, multimodal fusion meant combining various senses. The use of more than one input channel (e.g., gesture, speech, visual, haptic, etc.) to communicate with a machine in a system is referred to as multimodal interaction. As a result, adopting multimodal input interaction in virtual-reality settings might improve the naturalness and efficiency of interaction compared to using unimodal [8].

Ismail et al. [9] merged voice and gestures in the context of virtual-reality interaction, making the user's operation of dealing with virtual items in an AR environment more natural. Kadavasal and Oliver [10] created a virtual-reality driving system for autistic individuals that included physiological signals, brain signals, and eye gaze information, to improve autistic patients' driving abilities. Due to the lack of practical utility and popularity of virtual-reality experimental teaching, Xiao et al. [11] developed a multimodal interaction model that integrates voice and sensor information. Liu et al. [12] proposed a deep learning-based multimodal fusion model that combines three modal data sets: voice commands, hand gestures, and body movements, using various deep neural networks. In the field of automatic driving, Hu et al. [13] introduced contrastive learning approach to train a feature extractor with good representation ability in order to improve driving performance and avoid possible fatal accidents. In the field of speech recognition, Ondas et al. [14] proposed a combination of modified LIMA framework and iterative spectral subtraction algorithm to improve the robustness of speech recognition in noisy environment.

In short, the multimodal fusion interactive approach can solve the problems of input incomprehension, incompleteness, and misunderstanding caused by unimodal interaction with the system, as well as the ambiguity caused by relying on only one modality's input information to understand the user's intent. Existing multimodal fusion algorithms, on the other hand, primarily use a variety of single-channel information to assess user intent serially, but they are unable to fuse multichannel information to analyze user intent in parallel.

### 2.3. Smart Glove

The interaction method of keyboard and mouse combination has the attribute of having a weak sense of genuine operation in the field of human-computer interaction. Using traditional indoor interactive input devices for behavior detection, on the other hand, will result in the problems of occlusion and inability to observe subtle experimental phenomena. As a result, the above two human-computer interface methods each have their drawbacks. However, the advent of the smart glove gives a more natural human-computer interaction tool. It can interact with real objects using its presence of sensing devices and is not limited by the camera's field of vision, and it has responsiveness, good real-time, and high precision. Therefore, the smart glove is widely employed in a variety of applications, including sign language recognition, robot manipulation, and rehabilitation training.

Lokhande et al. [15] developed a glove that uses flex sensors and attitude sensors to transform gestures into text format in the field of conducting sign language recognition. Abhijith Bhaskaran et al. [16] created a smart glove that can recognize behaviors, gather hand position data, and transform recognized sign language into speech broadcast. Kumar Mummadi et al. [17] integrated an IMU module inside the glove's fingers, and the experimenter detected the wearer's hand posture and motion trajectory to perform French sign language recognition.

In the realm of robot manipulation and rehabilitation, the smart glove has also played an essential role. To enable remote manipulation of a robot, Roy et al. [18] designed a glove with flex sensors. Ma et al. [19] developed a smart glove to assist patients with weak hands in performing certain actions based on the direction and strength of fingertip movements during the rehabilitation process. Liu et al. [20] created a smart glove that uses inertial and magnetic measurement unit sensors to reconstruct hand movements accurately. Ge et al. [21] created a data glove that uses flex sensors to forecast the final gesture at the conclusion of the user's hand motion in real time.

In conclusion, the current virtual experiment platform primarily completes experiments by playing virtual animations, and users lack real-world experience with the platform. Simultaneously, other experiments rely primarily on unimodal interaction, which lacks an effective understanding and feedback of user intent. Although a traditional data glove can collect user data, it lacks knowledge of the user's intent and perception of external information during human-computer contact, and most of them only employ a single channel for interaction. Therefore, this paper proposes a smart glove with cognitive capabilities based on multimodal fusion, in which a multimodal fusion algorithm fuses user intent from visual, sensor, and voice channels in parallel at the intention level. The smart glove system can also employ MR technology to show the corresponding experimental phenomena and achieve the functions of guidance and error correction for the user after acquiring the user's final experimental intent.

## 3. Design of Virtual-Reality Fusion Experimental System Based on Smart Glove

### 3.1. Hardware Equipment

**Figure 1:**
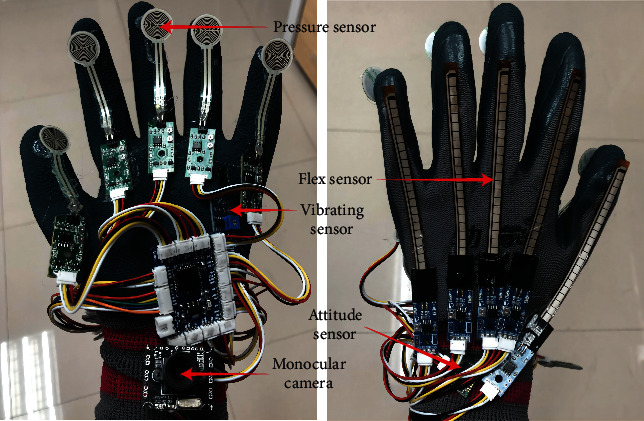
A physical prototype of the smart glove.

This paper designs a new smart glove with multisensing fusion in the context of a secondary school experiment. This smart glove can collect multimodal data about the user and then detect the user's operational intent, which is characterized by convenience, operability, and flexibility.  Figure 1 depicts a physical prototype of the smart glove.

Flex sensors, attitude sensors, pressure sensors, vibration motor modules, a monocular camera in the wrist part of the glove, and an indoor binocular camera applied to an MR experimental system make up the hardware element of the smart glove system. Different sensors can gather different information about the user's hands. To improve the traditional data glove which seriously affects the user's operation due to the complicated wires, this paper improves the data transmission between the smart glove and the computer to Bluetooth wireless transmission mode. The following is the functional design of each of its components.

1. Flex sensor: This sensor (Flex Sensor 4.5) is positioned in the smart glove's finger section and is used to obtain the degree of bending of the user's finger. The smart glove can map the bending changes of the sensor to the virtual hand in the Unity virtual scene, which is then utilized to restore the user's finger's bending state in real time.
2. Attitude sensor: This sensor (MPU6050) is positioned in the back of the smart glove's hand and is used to restore the user's hand's real-time posture by measuring the three-axis angle, velocity, and acceleration of the user's hand movement.
3. Pressure sensor: This sensor is located at the end of the smart glove's finger and is responsible for monitoring the user's finger end movements during operation.
4. Vibration motor module: The sensor is positioned in the back part of the smart glove's hand, and it can provide vibration feedback when the user grasps a real or virtual object.
5. Monocular camera: The camera is fixed in the wrist part of the smart glove, which can obtain the information of objects in experimental scene in real time and observe the subtle experimental phenomena. Its appearance solves the occlusion problem of traditional indoor input devices when identifying objects.
6. Indoor binocular camera for virtual-reality fusion: This camera is different from the occlusion-causing indoor camera. This binocular camera and Unity's Vuforia plug-in are used by the MR experimental system to provide a conduit between the actual world and the virtual world, allowing real experimental objects to interact with virtual experimental objects. Simultaneously, it can track the smart glove's real-time motion trajectory.

Trajectory Acquisition of Smart Glove Based on SGBM and YOLOv5 Fusion

During the experiment, the smart glove needs to acquire the user's hand movement trajectory and map it to the virtual experiment scene built by Unity in real time. The purpose is to enable the smart glove to interact with the experimental objects in the virtual-reality fusion experimental scene, which is the foundation for determining the user's experimental intent.

This paper incorporates the indoor binocular camera's binocular range function into the YOLOv5 target recognition procedure, while the smart glove is moving. The depth coordinates of the smart glove are obtained using the binocular stereo matching algorithm SGBM, with camera calibration, stereo correction, stereo matching, and range as the primary experimental steps. This fusion technique allows YOLOv5 to obtain not only the smart glove's position in the plane direction (i.e., *x*-axis and *y*-axis), but also the smart glove's depth value *z*, which is one of the paper's more difficult issues.

**Figure 2:**
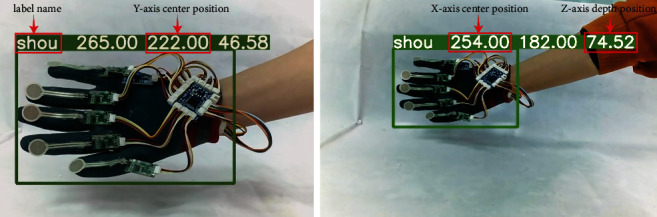
The three-dimensional position of the glove comparison picture (far and near position).

In this paper, the position of the indoor camera is used as the coordinate origin to obtain the 3D position of the smart glove in real time, as shown in Figure 2.

The coordinate information is processed using equation (1) based on the coordinate mapping relationship between the virtual scene and the camera position once the user's hand movement trajectory is collected. It is worth mentioning that (Pos_*x*_, Pos_*y*_, Pos_*z*_) is the smart glove's 3D position mapped to the virtual world, and *k* is the coordinate transformation's scale factor. Finally, the data are transferred to the Unity platform through a socket connection.

(1) $$\begin{array}{c} \left[\begin{array}{c} {\text{Pos}}_{x}\\ {\text{Pos}}_{y}\\ {\text{Pos}}_{z} \end{array}\right]=k\left[\begin{array}{c} x\\ y\\ z \end{array}\right]. \end{array}$$

### 3.2. MR Experiment Scene Building

The majority of virtual laboratories are provided in the form of augmented reality (AR) or virtual-reality (VR), with participants immersed in a purely imaginary scenario of the experiment without being able to observe their actions in detail. As a result, this paper uses Unity's Vuforia plug-in and an indoor binocular camera to create an MR lab. This lab can integrate virtual sceneries and the actual world using AR technology, allowing them to cohabit and interact. MR is divided into two components in its practical application. The virtual object exhibited outside the screen is one component, and the real scene displayed inside the screen is the other.

**Figure 3:**
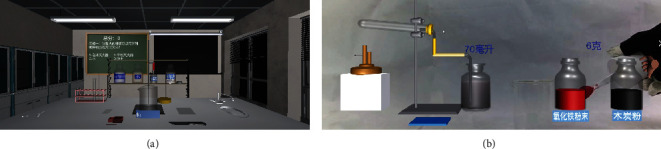
Comparison between traditional AR chemistry laboratory and MR chemistry laboratory.

The footage from the indoor camera is combined with the virtual world, creating a new visualization of the experimental environment. This fusion process allows users in the MR lab to genuinely watch their operations as well as observe the experimental phenomena that arise as a result of those operations as shown in Figure 3.

The smart glove system incorporates a speech recognition module to better conduct the virtual-reality fusion experiment teaching and collect the user's intent. The Baidu voice SDK is used in this study to continually monitor and recognize the user's voice information. And the MFA is used to calculate the intention probability after the effective information in the experiment is obtained; at the same time, the module uses the Unity's SpVoice plug-in to provide users with voice guidance or explain experimental phenomena, reducing the user's memory burden.

## 4. Design of Intelligent Cognitive Module

### 4.1. Overall Framework

**Figure 4:**
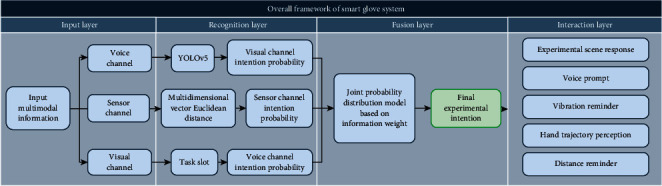
The overall framework of smart glove system based on multimodal fusion.

To guide and correct users' experimental operations, this paper uses the cognitive-behavioral theory [22], which refers to altering bad cognition by modifying the user's thinking and behavior. Users' thinking and actions should be tightly interwoven in the ideal situation. However, in real-world situations, users' cognition formation will be influenced by their automatic thinking; i.e., some users' actions will perform certain incorrect operations without thinking about it. As a result, the development of cognitive behavior theory can assist users in rationally correcting their unthinking conduct. Based on this, this paper constructs a general framework of a smart glove system based on multimodal fusion, as shown in Figure 4.

In the input layer, the smart glove acquires multimodal information from the user's voice, visual, and sensor channels, and passes the data into the recognition layer. The recognition layer and the fusion layer are critical components of the smart glove's overall framework. Under the background of intelligent experiment, the TMFA has the flaw of user experiment intention obtained by serial fusion of single-mode information. To overcome this challenge, the intent probability models for the voice, visual, and sensor channels in the recognition layer are established, and they are utilized to update the intent probability of their respective channels in real time. By studying user behavior, these intent probability models convert the abstract intent of user behavior under each channel into a computable set of intent probabilities by analyzing the user behavior. In the fusion layer, this study ingeniously proposes the MFA, which dynamically updates the associated weight of each channel using the information weight method, fuses the intent probability set produced by the recognition layer in parallel, and finally determines the user's experimental intent.

In summary, the essence of the model and algorithm proposed in this paper is to convert the abstract intent, which is difficult to speculate, into a mathematical language that can be computed.

### 4.2. Model of Visual Channel Based on YOLOv5

The monocular camera on the smart glove can perceive the entire experimental scenario through the visual channel. To obtain the user's experimental intent under the visual channel, this model uses the incremental change of the bounding box area of the experimental object in YOLOv5 to infer the probability of the user's experimental intent. The target object obj_*i*_ will be dynamically updated each time the user performs an experimental operation. The visual channel intent probability acquisition algorithm (hereinafter referred to as VSIPAA) is based on YOLOv5, as shown in Algorithm 1.

Different experimental items correspond to one or more experimental intent in the visual channel. When the VEIPAA algorithm updates the usage probability of the experimental items, the probability of one or more experimental intents corresponding to them is also updated in real time.

### 4.3. Probability Model of Sensor Channel Based on Euclidean Distance

Distinct experimental actions correlate to different experimental intents in the sensor channel. During the experimental operation, the user dynamically generates a huge amount of complex sensor data, so this paper sets up a seven-dimensional vector *γ* to establish the mapping relationship among the three flex sensors, pressure sensors, and attitude sensors. On this basis, this paper designs the Euclidean distance sensor channel based intent probability acquisition algorithm (hereinafter referred to as SRIPAA) in the seven-dimensional space, as shown in Algorithm 2.

Under the sensor channel, different actions correspond to one or more experimental intents, and when the probability of the current user action is updated by the SRIPAA algorithm in this paper, the probability of the corresponding one or more experimental intents behind it is also updated simultaneously in real time.

### 4.4. Probability Model of Voice Channel Based on Task Slot

**Figure 5:**
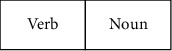
Task slot.

Users can enter speech information at any point during the experiment in the voice channel. This study divides speech_*i*_ into verb *s*_*v*_ and nouns *s*_*n*_ using Baidu voice recognition and lexical analysis technology, and put them into the task slot to get the instruction *V*, as shown in Figure 5. Simultaneously, the voice command *V* performs similarity matching with the system speech library SPEECH task slot.

Under the voice channel, this paper designs a voice channel intention probability acquisition algorithm based on task slot (hereinafter referred to as VCIPPA), as shown in Algorithm 3.

### 4.5. Multimodal Fusion Algorithm

Multimodal fusion refers to the overall merging of all input information before the system confirms the user's intent in the context of a virtual-reality fusion experiment. The TMFA completes the experiment by applying the rule that one channel's input information corresponds to one experimental intent, which essence is that the serial integrates the diversity of single-mode information, rather than the parallel fusion of all modal intent at the intention level.

Therefore, this paper inventively proposes MFA based on the smart glove system, which calculates the coefficient of variation of each channel using the information weight method and obtains the weights of each channel by normalization, and then the joint intent probability of multimodal fusion is calculated and generated and achieves the function of fusing user multimodal intent information in parallel on the intent layer in the following steps:

### 4.6. Algorithm Analysis

Algorithm validity means that when the input value satisfies the condition, the algorithm should work properly and output the corresponding result; i.e., whether the breakpoint is set in the voice channel, the visual channel or the sensor channel will output the corresponding intent. In the real experiment, the user may not input the information of three channels at the same time; for example, the user may not input the voice during the operation. In that case, the algorithm will update the coefficient of variation of the visual and sensor channels in real time and flexibly to calculate the final intent probability.

When the user perceives the experimental scene information using the smart glove's camera on the wrist, the MFA employs YOLOv5 to identify the experimental objects in the scene and calculates the increase or decrease of the area of the experimental object between two frames in real time. This algorithm feeds the area increment change value of the experimental object's bounding to the VSIPAA algorithm, and the probability set Visual of the experimental intent is obtained under the visual channel.

When users use the smart glove to operate real or virtual experimental objects, the smart glove will continuously generate a large amount of unavailable and independent complex data in the process of operation, so the SRIPAA algorithm sets up a seven-dimensional vector *γ* to establish the mapping relationship between the three sensors, so the intent inference problem of the sensor channel can be converted into a problem of calculating the distance between vectors in the high-dimensional space. To acquire the experimental intent probability set Sensor under the sensor channel, the MFA utilizes SRIPPA algorithm to calculate the Euclidean distance of the vector in the high-dimensional space.

Under the voice channel, users can input different speech information speech at any time. And the MFA utilizes VCIPAA algorithm to generate input speech task slot *V*. By matching with the similarity of system speech library SPEECH, the intent probability set Voice under the voice channel can be easily calculated.

Throughout the experiment, the MFA changes the value of each channel's intent probability set in real time, as well as the corresponding weight value, i.e., the coefficient of variation, in response to the change in the probability value. Finally, based on the normalized coefficients of variation, the MFA performs an intention-level fusion of the probability sets of the three channels to produce the user's final intent.

The MFA efficiently handles two theoretically suggested problems in this paper: (1) the MFA provides an unobstructed real-time perception of scene information using the visual channel at the wrist of the smart glove compared to the indoor interactive input devices; (2) the MFA can fuse the user's multimodal intent in parallel at the intention level and improves on the TMFA serial processing of modal information.

## 5. Analysis of Experimental Results

### 5.1. Setting

The computer used to conduct the research in this paper had an i7-10875H processor with a 2.30 GHz processor and 16 GB of RAM. The virtual-reality fusion lab was designed using Unity with version number 2018.3.8. To evaluate the effectiveness of the smart glove system with multimodal fusion in improving the teaching of MR experiments, 30 volunteers were invited to participate in the experiments while designing the comparison experiments in this paper. The volunteers included 10 elementary school students and 20 secondary school students with a male-to-female ratio of 1 : 1. The ages of these students were concentrated between 8 and 16 years old, and none of them had ever used the smart glove MR experiment system.

### 5.2. Experiment: Reduction of Iron Oxide by Charcoal

**Figure 6:**
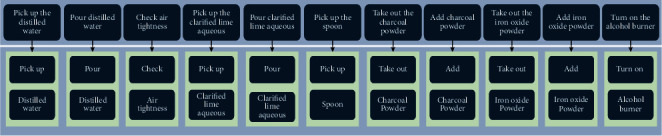
System speech library.

In this paper, a multimodal fusion of smart glove was utilized for the charcoal reduction of iron oxide experiment. And the system speech library of this experiment is shown in Figure 6. There are 11 user intentions in the whole experimental system: pick up the distilled water (*I*1), pour distilled water (*I*2), check air tightness (*I*3), pick up the clarified lime aqueous (*I*4), pour clarified lime aqueous (*I*5), pick up the spoon (*I*6), take out the charcoal powder (*I*7), add charcoal powder (*I*8), take out the iron oxide powder (*I*9), add iron oxide powder (*I*10), and turn on the alcohol burner (*I*11).

Meanwhile, according to the experimental requirements, this paper sets up the experimental intent library to analyze the user's experimental intent, as shown in Table 1.

**Figure 7:**
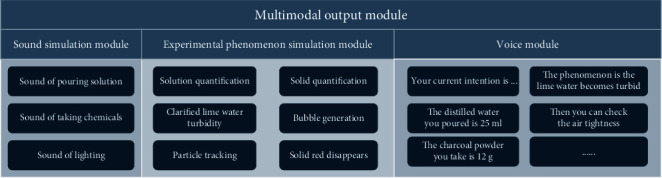
Multimodal output module.

The smart glove system receives the user's multimodal data in real time and converts it into the intent probability set of each channel throughout the user experiment operation. Next, Algorithm 4 performs a parallel fusion of intention levels on the intent probability set, before finally displaying the user's current experimental intent. As illustrated in Figure 7, this study establishes a multimodal output module [23] based on phenomenon visualization and speech output according to user intention information. After obtaining the user's intent, the module simulates the corresponding experimental phenomena, and guides and corrects the essential steps or incorrect steps to help the user through the experiment.

**Figure 8:**
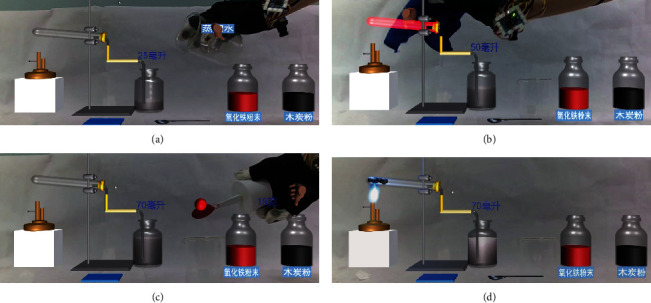
(a) A diagram of the user wearing a smart glove to pour distilled water. (b) A diagram of the user picking up a hot towel for gas tightness test. (c) A diagram of the user removing the iron oxide powder after the grains follow the movement of the tip of the spoon. (d) A diagram of the experimental phenomenon after the user ignites the alcoholic blowtorch.

In this experiment, the second step is to pour distilled water. The user wears a smart glove, pours liquid from a real beaker into a virtual beaker, and inputs the voice “pour distilled water.” Through Algorithm 4, the system deduces that the user's present behavior intends to “pour distilled water.” The MR experiment system gives real-time feedback on the user's current behavior through the experiment scene animation, as shown in Figure 8(a). When the smart glove senses the user taking up the hot towel and covering the virtual beaker's wall, the system may still utilize Algorithm 4 to infer that the user's present behavior is “check air tightness” even without the voice input of “check air tightness.” The experiment uses information augmentation technology to display the bubbles formed in the virtual beaker to demonstrate that the apparatus is well gasketed, as shown in Figure 8(b). As the operation proceeds, when the smart glove detects that the user picks up the medicine spoon to take chemicals from the fine mouth bottle containing iron oxide powder and enters the voice “take out iron oxide powder,” the smart glove system obtains the current operation behavior of the user as “take out the iron oxide powder.” The virtual iron oxide powder pellet in the experimental system follows the tip of the medication spoon, as shown in Figure 8(c). In the last step, after the user places the iron oxide powder and charcoal powder into the test tube, operates the virtual alcohol blowtorch, and at the same time enters the voice “light the alcohol blowtorch,” the system will get the current behavior of the user as “turn on the alcohol blowtorch.” As shown in Figure 8(d), the MR experiment system shows the virtual flame of the alcohol torch burning and the clarified lime water turning cloudy.

### 5.3. Speech Instruction Verification

The smart glove system can evaluate various speech information of users for voice input. The VCIPAA algorithm translates the user's speech information into instruction *V* and performs similarity matching with the system speech library, so as to update the intent probability set of the voice channel. Although users can input varied speech information into the smart glove system at any time, the intent probability of voice channel will change only when they input speeches similar to the system speech library. Consequently, to verify the stability of the voice channel, a comparative experiment is set up and 10 volunteers are invited to input the 11 instructions of the system speech library and irrelevant instructions, and the consequences of their recognition accuracy are tallied, as shown in Table 2.

Instead of wearing smart glove throughout the recognition process, the user can only input speech information, preventing information from other channels from interfering with the verification of voice channels. It should be highlighted that a successful recognition instruction indicates that the MR experimental system will display the related experimental phenomenon when the user inputs the corresponding speech.

As shown in Table 2, the MR system will not respond if the user enters irrelevant instructions. However, the recognition accuracy reaches 97.27% when users input the relevant instructions of the system speech library, but it is also affected by network delay or nonstandard user pronunciation. To summarize, the speech channel has a high level of stability, which is one of the foundations for MFA accurately identifying the user's experimental intent.

### 5.4. Occlusion Handling

**Figure 9:**
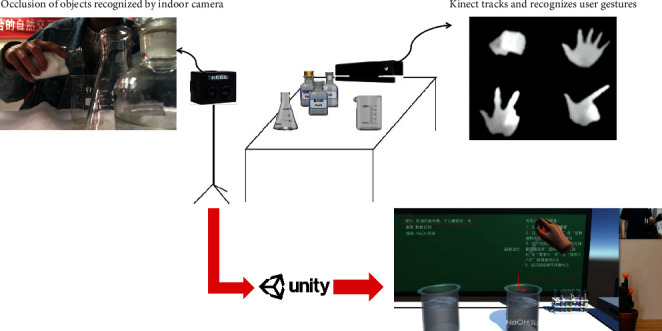
Diagram of the occlusion problem.

One of the biggest issues with traditional indoor interactive input devices in the context of intelligent experiments is occlusion. The instruments and devices in the experimental scenario are often occluded due to different placements, making the input device unable to perceive and recognize key information, especially difficult to recognize tiny objects and observe subtle phenomena. Traditional virtual-reality fusion chemistry experiments [2] used KINECT for user gesture channel information acquisition and a binocular camera for visual channel information acquisition. As a result, it is easy to have a problem during the experiment with improper recognition or occlusion of experimental objects and gestures, which impacts the impression of the user's intent, as shown in Figure 9. Therefore, this paper uses the camera at the wrist of the smart glove for proximity perception of the scene to solve the problems existing in the traditional virtual laboratory.

**Figure 10:**
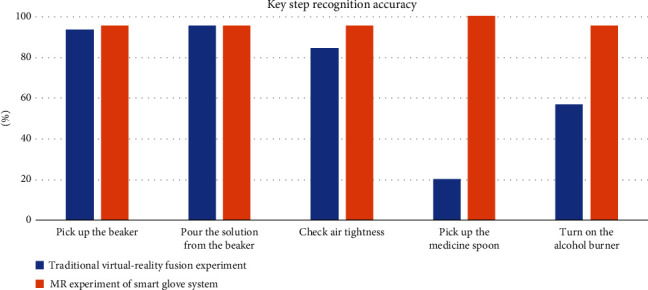
Key step recognition accuracy.

In this paper, 30 volunteers were invited to participate in a comparative experiment in which they completed several key steps of both the traditional virtual-reality fusion experiment and the MR experiment based on smart glove system. Each volunteer conducted 3 times, their recognition accuracy was counted (the recognition success here means that the virtual-reality fusion platform can show the correct experimental phenomenon), and the results are shown in Figure 10.

As can be learned from Figure 10, compared with the traditional virtual-reality fusion experiment, the smart glove system has a significant advantage in terms of observation of tiny objects, subtle movements, and minute phenomena.

For the action of picking up a medicine spoon, it is difficult for KINECT to respond to it because the spoon is small and not easy to recognize, but the wrist camera of smart glove system can easily recognize the object in the experimental scene. For the action of turning on the alcohol burner, the magnitude of the action is small, so the KINECT device can easily recognize the gesture as other actions. However, the smart glove system can employ MFA to fuse multichannel information to recognize the action.

In summary, the smart glove system can solve the problem that it is difficult to cope with subtle actions and micro-phenomena in the traditional virtual-reality fusion experiment.

### 5.5. Verification of Multimodal Fusion

Users can complete the MR chemical experiment by using a single-mode interaction with an independent voice channel or by combining two or three modes. However, during the experiment, the visual and sensor channels will continuously collect data from the user, while the voice channel will be input according to the user's choices, allowing the user to choose the number of channels to employ based on the current experimental steps.

In order to verify that multimodal fusion can better identify user experimental intent than single mode, 20 volunteers were invited to complete the experiment 4 times using (1) voice channel, (2) dual-mode fusion of visual channel and sensor channel, and (3) multimodal fusion of three channels, and counted the relationship between the average completion rate, average completion time (in seconds), and user satisfaction (10-point scale) to verify that multimodal fusion can better identify users' intentions than single-mode fusion. (The completion experiment here means that the MR platform displays the operation's reaction outcomes in real time.) Table 3 displays the validation findings.

Table 3 can be used to draw the following conclusions:

1. When the user uses the voice channel alone, the experiment completion rate is 97.5% since the input voice information is rather constant. Users, on the other hand, are unable to operate genuine experimental objects, which result in the user experience is terrible.
2. Because an experimental object in the visual channel and an action in the sensor channel might both correspond to separate experimental intentions at the same time, the average completion rate drops, but the user's score improves significantly when compared to the single voice channel. Because the data from the sensor and visual channels are complicated, the temporal complexity of dual-mode fusion increases, resulting in a longer average completion time for users.
3. Due to the nonstandard operation of the user in the multimodal fusion process, the average completion rate of the experiment will be lower than that of utilizing only the voice channel, but the user score will be the highest in the comparative experiment. Furthermore, when compared to dual-mode fusion, after the addition of a voice channel, the average completion time of a multimodal fusion experiment is lowered. So, it is shown that the experimental interaction mode of multimodal fusion can suit users' needs and can better comprehend their experimental intentions.

### 5.6. Verification of MFA Algorithm

**Figure 11:**
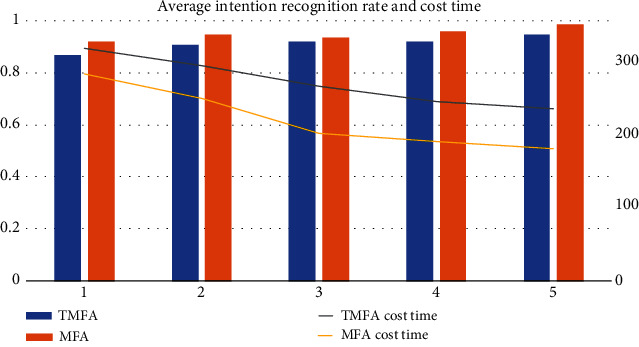
Comparison between MFA and TMFA.

Although the TMFA [11] uses multichannel data information in the experimental process, its essence is serial fusion of multimodal information, which means that only one channel of information is used in each intent recognition. The essence of MFA is the parallel fusion of multimodal intent probability. In order to verify if the MFA has a superior intent recognition impact than TMFA, 15 volunteers were invited to complete experiments of iron oxide reduction with charcoal 5 times using the two algorithms in a comparison study.  Figure 11 shows the average intention recognition rate and the cost time (in seconds) of the experiment.

Figure 11 shows that the average intent recognition rate of the two algorithms in each experiment is more than 90%, demonstrating that the two algorithms can accurately identify the user's intention and that the user's cost time to complete the experiment with the help of the two algorithms decreases as the experiment progresses. However, when compared to the TMFA, the MFA has a higher average intent recognition rate and reduces the time to complete the experiment by around one minute, demonstrating that the MFA can assist users in accurately and efficiently completing the experiment, and can be applied to middle school chemistry experiment education.

### 5.7. Cognitive Burden Assessment

**Figure 12:**
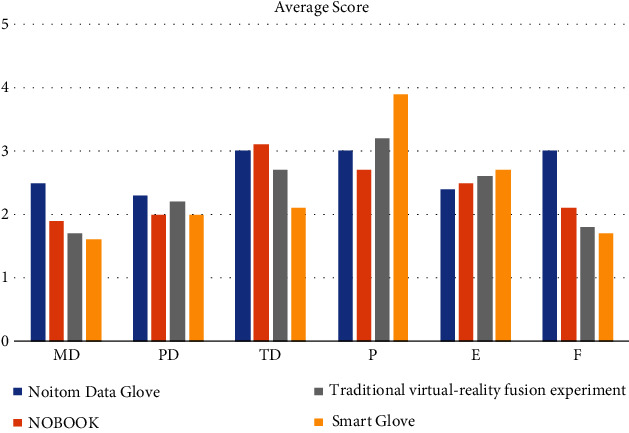
NASA user evaluation.

To highlight the ability of the smart glove to perceive the behavioral intent of users, a comparison experiment was designed in this paper. First, volunteers conducted experiments on a virtual experimental platform using the Noitom data glove [24] and the KINECT device; second, volunteers perform the same experiment on the NOBOOK platform [1], which is dominated by keyboard and mouse operations; third, volunteers use the traditional virtual-reality fusion experimental platform [2] to conduct experiments; at last, volunteers perform the same experiments using the smart glove. 30 volunteers were required to take turns in conducting experiments on the above four experimental platforms during a day and to perform NASA evaluation [25] of each experiment after completion. User evaluation metrics were categorized into mental demands (MD), physical demands (PD), time demands (TD), performance (*P*), effort (*E*), and frustration (*F*). The NASA assessment metrics were evaluated on a 5-point scale. 0 to 1 indicates a low cognitive burden, 1 to 2 indicates a relatively low cognitive burden, 2 to 3 indicates an overall cognitive burden, 3 to 4 indicates a relatively high cognitive burden, and 4 to 5 indicates a very high cognitive burden. The results are shown in Figure 12.

Figure 12 shows that the MD and TD index scores of smart glove are lower than those of other platforms, indicating that the experimental process of smart glove is simpler than that of others. This is because when using other platforms for experiments, volunteers need to understand the various functions of the platform in advance, such as the construction of the NOBOOK experimental platform. The smart glove system and the virtual-reality fusion experimental platform scored higher on the P index. When it came to running the experiment, volunteers indicated they spent the majority of their time learning how to utilize other two platforms. When they wear smart glove for the experiment, they pay more attention to the phenomenon and results of the experiment. The experimenter can deepen their understanding of the experimental phenomenon by observing the phenomenon on the screen and the system's explanation of the experimental mechanism. At the same time, the smart glove system will also correct the nonstandard behavior in the experimental process, so that they will have a deeper impression of the key points of the experimental operation.

In conclusion, as compared to existing experimental platforms, the smart glove system developed in this paper allows users to conduct experiments more intelligently and naturally, as well as improves users' experimental immersion and operation ability.

## 6. Conclusions

Some middle school experiments currently have issues with powerful destruction and expensive costs, while the traditional experimental platform suffers from a poor sense of operation and a significant memory burden. As a result, this paper develops a smart glove using the MFA to detect the user's experimental intent and then direct the user to experiment, or correct and remind the user's incorrect and harmful actions.

To address the aforementioned issues, this study primarily performs the following two original contributions:

1. This study designs and implements a smart glove system. The smart glove can address the issues of (a) the lack of cognitive ability of traditional data gloves and (b) the occlusion phenomenon caused by too many indoor interactive input devices in the traditional virtual-reality fusion chemistry experiment. The traditional data glove can only gather the user's hand data and cannot capture the user's experimental intent; thus, several indoor interactive input devices are required to aid the user during the perception process of scene information in real time, which can easily cause occlusion in object recognition. However, the smart glove system can realize the observation of tiny objects, subtle movements, as well as minute phenomena, increase interaction efficiency, and decrease memory pressure. However, the smart glove's hardware arrangement is unreasonable, and it is difficult to wear when the user's palm is large; the MR system is currently just for chemical research. As a result, future research should focus on improving the smart glove's structure and expanding the experiment library so that users can complete a wide range of experiments in order to achieve the goal of virtual-reality education [26].
2. A parallel MFA integrating sensor, voice, and visual channels is proposed in this paper to address the problem that the TMFA only integrates user modal information in serial. The essence of TMFA is the serial fusion of users' multimodal information, and it does not support the simultaneous analysis of users' intentions using multimodal data. However, the MFA is a multimodal fusion algorithm based on multichannel intention probability fusion, which can accurately and efficiently obtain the user's intent and guide user's behavior based on that intent. Based on the user's intent, future research should be devoted to predicting the user's intent in the following stage and building a human-computer cooperation model so that the user can wear the smart glove and cooperate with the computer to complete experiments.

## Figures and Tables

**Algorithm 1:**
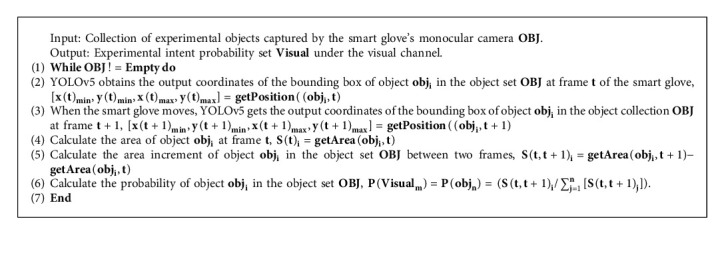


**Algorithm 2:**
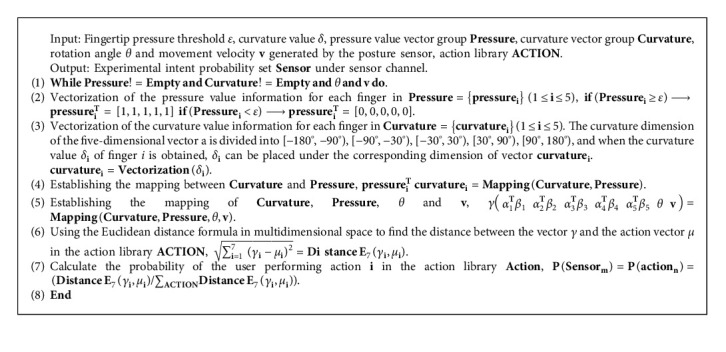


**Algorithm 3:**
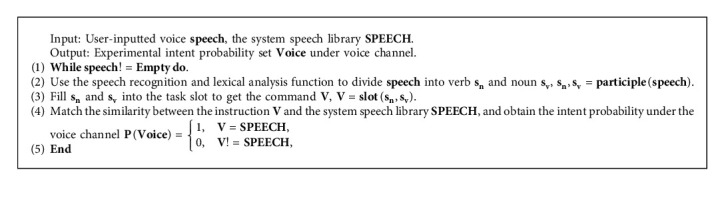


**Algorithm 4:**
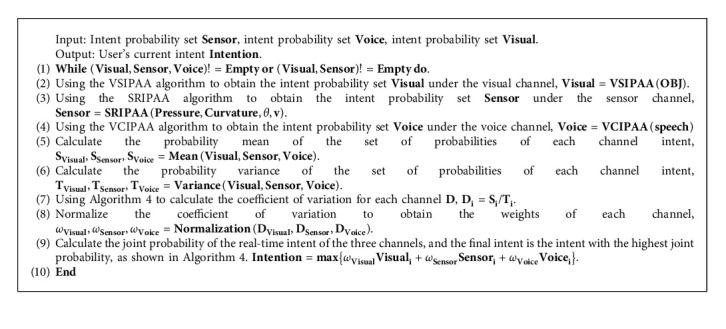


**Table 1:** Experiment library under voice channel, visual channel, and sensor channel.

Intent	System speech library task slot	Corresponding recognition object	Corresponding recognition action
*I*1	[Pick up, distilled water]	Beaker with water	Pick up
*I*2	[Pour, distilled water]	Beaker with water	Pour
*I*3	[Check, air tightness]	Hot towel	Grasp
*I*4	[Pick up, clarified lime aqueous]	Wide mouth bottle with clarified lime water	Pick up
*I*5	[Pour, clarified lime aqueous]	Wide mouth bottle with clarified lime water	Pour
*I*6	[Pick up, spoon]	Medicine spoon	Pinch
*I*7	[Take out, charcoal powder]	Fine mouth bottle with charcoal powder	Pinch
*I*8	[Add, charcoal powder]	Fine mouth bottle with charcoal powder	Pinch
*I*9	[Take out, iron oxide powder]	Fine mouth bottle with iron oxide powder	Pinch
*I*10	[Add, iron oxide powder]	Fine mouth bottle with iron oxide powder	Pinch
*I*11	[Turn on, alcohol burner]	—	Poke

**Table 2:** Relevant instructions and irrelevant instructions.

	Relevant instructions	Irrelevantinstructions
Total times	110	110
Effective times	107	0
Recognition accuracy	97.27%	0%

**Table 3:** Comparison of three methods.

Intent name	Voice channel	Dual-mode fusion	Multimodal fusion
Average completion rate	97.5%	88.75%	95%
Average completion time	71	274	201
User satisfaction	3.9	6.05	8.9

## Data Availability

The data used to support the findings of this study are available from the corresponding author upon request.
